# Barriers and facilitators to effective electronic health record-based sepsis screening in the pediatric intensive care unit

**DOI:** 10.1093/jamiaopen/ooae048

**Published:** 2024-07-08

**Authors:** Stacey M Sears, Anisha K Coughlin, Kathryn Nelson, Terri Stillwell, Erin F Carlton, Heidi R Flori

**Affiliations:** Department of Pediatrics, Division of Pediatric Critical Care Medicine, University of Michigan Medical School, Ann Arbor, MI 48109, United States; School of Nursing, Wayne State University, Detroit, MI 48202, United States; Department of Pediatrics, Division of Pediatric Critical Care Medicine, University of Michigan Medical School, Ann Arbor, MI 48109, United States; University of Michigan School of Nursing, Ann Arbor, MI 48109, United States; Department of Pediatrics, Division of Infectious Disease, University of Michigan Health System, Ann Arbor, MI 48109, United States; Department of Pediatrics, Division of Pediatric Critical Care Medicine, University of Michigan Medical School, Ann Arbor, MI 48109, United States; Department of Pediatrics, Division of Pediatric Critical Care Medicine, University of Michigan Medical School, Ann Arbor, MI 48109, United States

**Keywords:** sepsis screening tool, quality improvement, pediatric intensive care unit, EMR, end-user experience

## Abstract

**Introduction:**

The Pediatric Surviving Sepsis Campaign supports the implementation of automated tools for early sepsis recognition. In 2019 the C.S. Mott Children’s Hospital Pediatric Intensive Care Unit deployed an electronic medical record (EMR)-based screening for early recognition and treatment of sepsis.

**Materials and Methods:**

We analyzed all automated primary sepsis alerts, secondary screens, and bedside huddles from November 2019 to January 2020 (Cohort 1) and from November 2020 to January 2021 (Cohort 2) to identify barriers and facilitators for the use of this tool. We distributed surveys to frontline providers to gather feedback on end-user experience.

**Results:**

In Cohort 1, 895 primary alerts were triggered, yielding 503 completed secondary screens and 40 bedside huddles. In Cohort 2, 925 primary alerts were triggered, yielding 532 completed secondary screens and 12 bedside huddles. Surveys assessing end-user experience identified the following facilitators: (1) 73% of nurses endorsed the bedside huddle as value added; (2) 74% of medical providers agreed the bedside huddle increased the likelihood of interventions. The greatest barriers to successful implementation included the (1) overall large number of primary alerts from the automated tool and (2) rate of false alerts, many due to routine respiratory therapy interventions.

**Discussion:**

Our data suggests that the successful implementation of EMR-based sepsis screening tools requires countermeasures focusing on 3 key drivers for change: education, technology, and patient safety.

**Conclusion:**

While both medical providers and bedside nurses found merit in our EMR-based sepsis early recognition system, continued refinement is necessary to avoid sepsis alert fatigue.

## Introduction

Sepsis accounts for 8% of admissions and up to 25% of all deaths in pediatric intensive care units (PICUs) around the world^1^ and increases the length of stay 8-fold compared to nonsepsis cases.^2^ Currently, automated pediatric sepsis recognition and screening tools, especially electronic medical records (EMRs) based, remain understudied.^3^

The 2020 Pediatric Surviving Sepsis Campaign (pSSC), a worldwide initiative by the Society of Critical Care Medicine (SCCM), and the European Society of Intensive Care Medicine (ESICM) has augmented awareness of the morbidity and mortality of sepsis in pediatric patients.^3^ The pSSC guidelines strongly recommend implementing systematic screening for early recognition of septic shock and other sepsis-related organ dysfunction.^3^^,^^4^ Additionally, the Children’s Hospital Association Improving Pediatric Sepsis Outcomes (IPSO) initiative, a collaboration of more than 55 Children’s Hospitals, endorses early sepsis screening and intervention practices and sharing successful methodologies among participating centers.^5^

Automated sepsis screening tools use data from the EMR to detect patients with sepsis across all areas of the hospital.^3^ In general, tools track systemic inflammatory response syndrome (SIRS) criteria such as heart rate, respiratory rate, temperature, and white blood cell count, based on the SCCM and the American College of Critical Care Medicine guidelines.^3^ Unfortunately, existing sepsis identification tools vary in framework, interface, and implementation, limiting the ability to meta-analyze the effects of successful use and thus generalizability across institutions.^6^ An ideal electronic sepsis detection tool should accurately predict sepsis with timely alerts, exhibit minimal variability, and seamlessly integrate with bedside provider workflow.^3^^,^^6^ At our institution, the IPSO task force created an EMR-based, primary sepsis alert using vital sign parameters to enable a highly *sensitive* screening tool.^7^^,^^8^ Sepsis was defined using IPSO’s “time zero” sepsis and severe sepsis, and septic shock definitions. Once triggered, a secondary, confirmatory screen is completed by the bedside nurse. If this secondary screen reaches the defined threshold, a multidisciplinary bedside huddle, involving a clinician review of sepsis criteria, occurs to more *specifically* identify those patients with sepsis and to institute timely confirmatory diagnostics and treatments.

In this assessment of a previously implemented quality improvement measure, we executed the first comprehensive assessment of this primary alert, secondary screen, and bedside huddle process since its initiation in September 2019 in our PICU over 2 time periods, November 2019—January 2020 (Cohort 1), and November 2020—January 2021 (Cohort 2) to assess the sustainability of the tool. Our project aims to identify barriers to and facilitators of appropriate and systematic identification of severe sepsis and septic shock patients and to understand key drivers allowing generation of potential countermeasures, or actions to further refine the PICU sepsis detection, screening, and response process.

## Methods

### Primary alert, secondary screen, bedside huddle process

**Figure 1.**
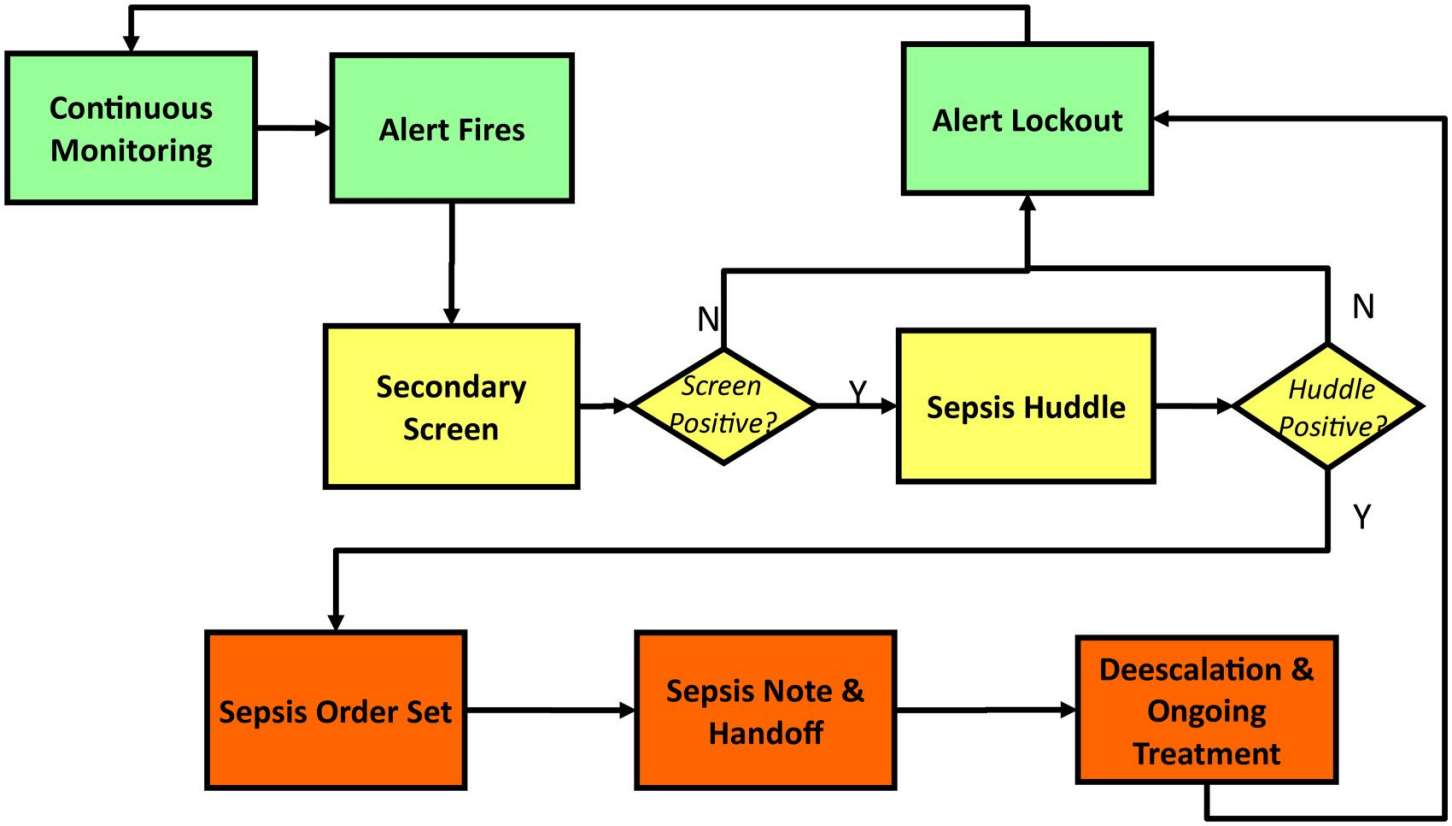
CDS work flow for the EMR sepsis trigger alert system as put together by Mott IPSO team in alignment with the Children’s Hospital Association.

Partnering with IPSO, our institution created a multidisciplinary team to develop and implement an EMR-based tool to systematically identify and treat patients with sepsis.^5^ Successful initiation of the tool on the inpatient acute care wards in April of 2019 generated background data to refine and create a tool directed to the PICU environment, given aberration in vital signs for age groups, including data to generate the primary alert trigger number (see below). In September 2019, the sepsis screening tool went live in the PICU. Figure 1 displays the workflow of the sepsis alert system adopted by our institution, the University of Michigan C.S. Mott Children’s Hospital (herein referred to as UM).

**Figure 2.**
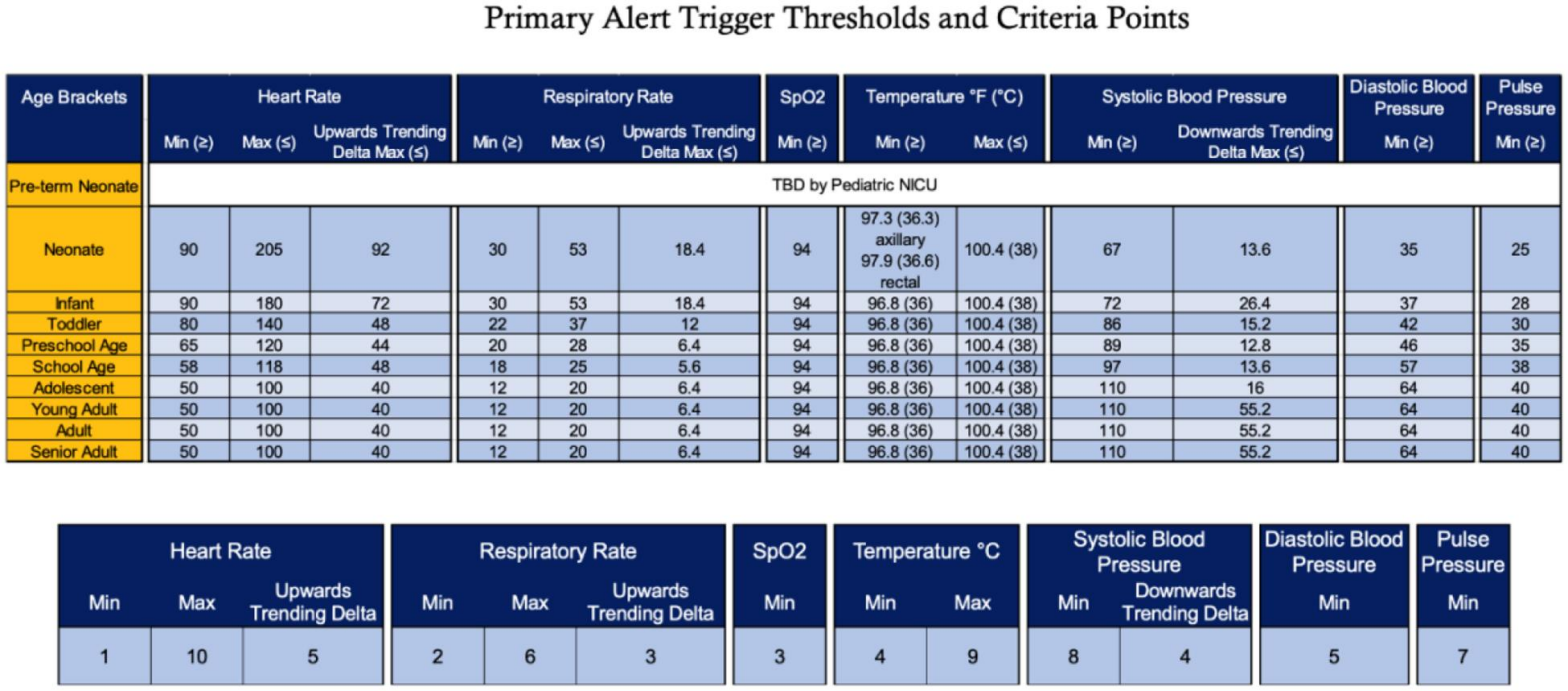
(Top) Parameters for primary alert trigger based on PALS guidelines. Please note that this chart represents the normal values, meaning that to add points in the rule logic, values must be less than the minimum or greater than the maximum thresholds seen. (Bottom) BPA triggers when the score is equal to or greater than 32. Please note that the way the rule logic is designed, points can be added for a criteria’s Min OR Max, but never both. However, for heart rate, respiratory rate, and systolic blood pressure, points can be added for (Min OR Max) AND Delta.

The primary alert triggered is designed to be highly *sensitive* to sepsis, identifying all potential patients who meet the criteria for sepsis. The primary alert utilizes age-specific vital sign parameters based on PALS, which are then verified in the EMR by any bedside provider who enters vital sign data into the medical record (eg, nurse or respiratory therapist). The primary alert assigns a numerical score for a change in vital signs, such as an increase or decrease in heart rate of at least 20% from the baseline. The trigger tool was allowed to run in the background of the EMR to establish trigger thresholds (28 for acute care patients and 32 for PICU patients) with appropriate sensitivity. When triggered, a primary sepsis alert activates a *best practice alert secondary screen* (hereafter called a secondary screen) (Figure 2). The medical providers can make additional modifications to the vital sign parameters to individualize threshold targets and prevent excess alerts and unnecessary bedside huddles if an aberration of certain vital sign parameters is expected.

At 32 points, the bedside nurse is required to complete an EMR *secondary screen.* The secondary screen includes 2 separate choices for the provider (1) *I’m not the patient’s nurse*, which will suppress the alert for 5 minutes, or (2) *I need more time,* which will silence the alert for 20 minutes. If the secondary screen is not completed on the initial alert, the subsequent alerts are considered a repeat, and the screen will reappear until completed to fill it out. If the bedside nurse does not complete the secondary screen within 15 minutes, the charge nurse will be automatically notified every 15 minutes until it is completed.

The *secondary screen* aims to specifically identify sepsis as the reason for the primary alert. This 7-question screen assesses changes in mental status, perfusion, respiratory status, urine output, laboratory values, family/caregiver concerns, or bedside provider concerns for sepsis with each question assigned up to 1 point except for bedside provider concern which could generate up to 4 points. A score of 4 or more out of 10 possible points constitutes a positive secondary screen. If at any time the bedside provider is extremely concerned the patient is clinically deteriorating due to sepsis, the nurse can respond “extremely concerned” on the secondary screen (score = 4), thus automatically generating a positive sepsis screen.

Once a positive secondary screen is met, a *multidisciplinary bedside huddle* is initiated. The EMR automatically pages the primary medical provider. Key clinician stakeholders, including the primary bedside nurse, primary medical provider (resident physician or nurse practitioner), and a respiratory therapist, if applicable, attend the bedside huddle. During bedside huddle, the team discusses the patient’s current condition and plan of care, using a sepsis order set, if needed. Finally, a sepsis note is placed in the patient’s medical record by the medical provider, documenting the occurrence of the bedside huddle, identification of sepsis, and any interventions performed.

If the bedside huddle confirms the clinical suspicion of sepsis (ie, clinician recognition and diagnosis of sepsis), the primary alert will lock out for the next 48 hours. If the secondary screen is negative (score of <4), there will be no bedside huddle, and the primary alert will lock out for 6 hours. Upon prompt response to the alert and completion of the huddle, the total time required was noted to be less than 15 minutes.

### Alert, screen, and huddle process evaluation

We utilized chart reviews and stakeholder surveys to identify barriers and facilitators to using automated sepsis alerts, secondary nurse screening and bedside huddles to improve care.

### Chart reviews

In collaboration with the IPSO task force and the Mott data analytics team, sepsis cases were identified by automated record pull and validated by IPSO-trained clinicians. IPSO defines sepsis by “stand-alone” criteria such as the use of a sepsis order set by the clinician or activation of a sepsis-specific huddle, or orders specifying sepsis-specific treatments such as antibiotics, fluid boluses, and/or vasoactive support.^6^ For 535 patient charts, we performed a comprehensive, manual chart review to identify barriers around responding to the primary automated sepsis alert, and the accuracy of the bedside huddle in effectively identifying pediatric patients with sepsis based on IPSO severe sepsis/suspected sepsis definitions.^9^ During variable extraction, reviewed data including vital signs, primary automated alerts, secondary nurse screens, and care team bedside huddles were captured.

### Stakeholder survey

We surveyed resident physicians, nurse practitioners, and bedside nurses about the IPSO program at Mott and the 2020 pSSC guideline recommendations to identify the barriers and facilitators associated with this process at the end-user level. One-time, 22-question Qualtrics^10^ surveys were emailed to all PICU nurses (*n* = 147), resident physicians (*n* = 79), and nurse practitioners (*n* = 10) in November 2020. Survey questions used multiple choice and Likert scale responses and addressed the following themes: education, technology, and patient safety.

Data were analyzed by using summary statistics in Excel (2021 version) and potential countermeasures were identified to further improve this systematic sepsis screening process. This study was deemed not regulated by the University of Michigan Institutional Review Board (HUM190779).

## Results

### Overall workflow data and chart review results—primary automated sepsis alert

**Figure 3.**
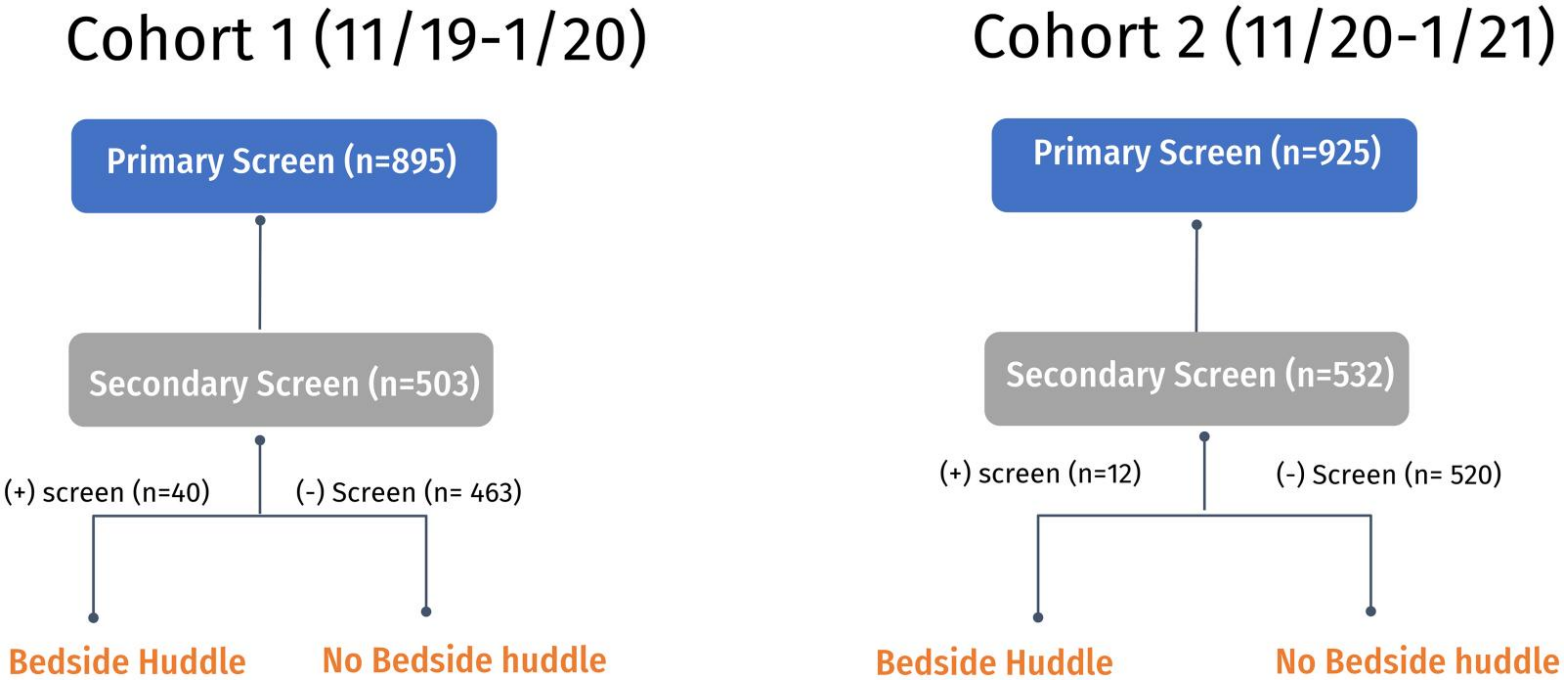
Cohort identification.

**Figure 4.**
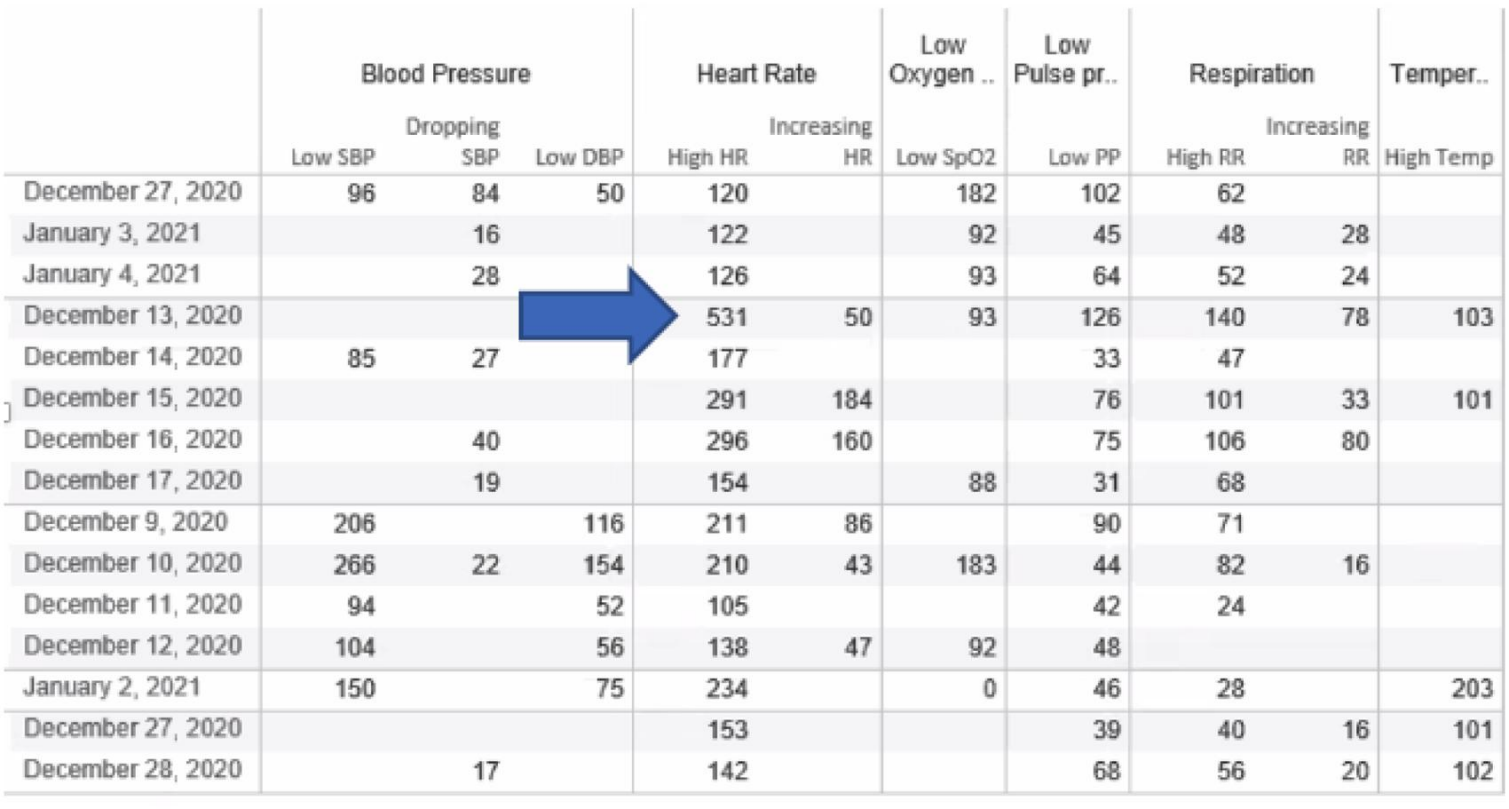
Example of documented vital signs, from cohort 1, which would trigger a primary screen. Note the blue arrow pointing to a documented Heart rate of 531. On chart review, Respiratory Therapy was performing chest physiotherapy via vest and validated the heart rate in the EMR.

From November 2019 to January 2020 (cohort 1), 895 primary automated sepsis alerts were triggered across 406 individual patients, of which 54% (*n* = 486/895) were repeats of the primary alerts. From November 2020—January 2021 (cohort 2), 925 primary sepsis alerts were generated across 140 individual patients with 51% (*n* = 473/925) repeats. Thus, there were multiple primary alerts per patient across both periods. Figure 3 displays cohort identification by using the steps of the sepsis pathway previously described. Overall, there was a mean of 9.7 initial primary alerts and secondary screens per day for the entire PICU population. Of the total alerts and secondary screens, 15% (*n* = 134/895) resulted from routine ancillary staff care activities (eg, following respiratory interventions such as breathing treatments, suctioning, or vest therapies) in the first cohort, and 12% (*n* = 114/925) for the second cohort (Figure 4).

### Secondary screen and bedside huddle

For Cohort 1, 895 primary automated sepsis alerts resulted in the completion of 503 secondary nurse screens. Of these, 8% (*n* = 40/503) scored “positive” by the bedside nurse with a screening score of 4 or greater, indicating the nurse was concerned about the potential for a sepsis diagnosis. In cohort 2, 925 primary automated sepsis alerts resulted in 532 completed secondary nurse screens, with only 2.2% (*n* = 12/532) being considered “positive” with a screening score of 4 or greater. All remaining secondary screens were deemed “negative” (score less than 4) indicating the EMR automated alert tool fired, although the bedside nurse did not assign a score high enough to trigger a bedside huddle.

### End user experience—nursing survey results

Among 147 eligible bedside nurses in the PICU, 92 (63%) completed the survey and identified facilitators of the sepsis identification and management process. Overall, (1) 72% of nurses agreed or strongly agreed the bedside huddle benefitted patient care, (2) 65% felt the overall process identified patients with sepsis appropriately, (3) 45% felt the process resulted in changes to patient care, and (4) 73% nursing survey respondents indicated the concerns of the bedside nurse were addressed during the huddle, and this was a value-added facilitator of the process.

**Figure 5.**
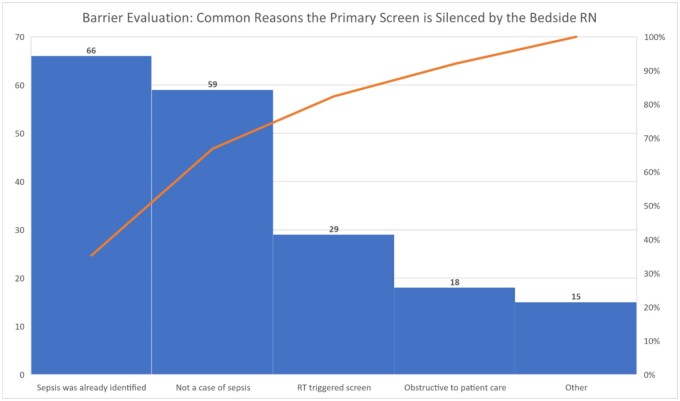
Pareto chart depicting barrier evaluation: common reasons the primary screen is silenced by bedside nurses.

The following barriers to effective sepsis screening tool implementation identified included: (1) lack of awareness of the pSSC guidelines given only 46% of bedside nurses were “definitely” or “probably” aware of the pSSC guidelines, (2) delay in writing of orders (70%; *n* = 64/92), (3) uncertainty by medical providers on choice of antibiotic to order (40%; *n* = 37/92), (4) pharmacy delays (96%; *n* = 88/92), (5) delays related to the first contact provider needing to discuss plans with the physician fellow or attending (59%; *n* = 54/92), and (6) need to consult pediatric infectious disease specialists (36%; *n* = 33/92) before antibiotic changes could be made. Figure 5 depicts a Pareto chart that further describes barriers for bedside nurses to silence the primary alert rather than completing the sepsis assessment.

### End user experience—nurse practitioner and medical resident survey results

Of 89 eligible resident physicians and/or nurse practitioners, 39 (44%) responded to the survey (8 NPs, 15 second-year pediatric residents, 13 third-year general pediatric residents, 2 medicine-pediatric residents, and 1 emergency medicine resident). In total, while 67% (*n* = 26/39), were definitely or probably aware of the 2020 pSSC guidelines, only 38% (*n* = 15/39), were definitely or probably aware of Mott partnering with CHA IPSO on the systematic sepsis process. When asked about confidence in identifying the stages of sepsis, the majority were somewhat confident: 59% (*n* = 23/39) in diagnosing SIRS, 56% (*n* = 22/39) in diagnosing sepsis, 54% (*n* = 21/39) in septic shock, 77% (*n* = 30/39) managing sepsis or septic shock. Survey questions evaluating the bedside huddle indicated 72% (*n* = 28/39) of providers reviewed the plan of care during the huddle, 67% (*n* = 26/39) performed a clinical assessment of the patient, and 38% (*n* = 15/39) performed an intervention. A minority, (21%, *n* = 8/39) of providers never attended a bedside huddle in the PICU. At the bedside huddle, 26% (*n* = 10/39) of the providers indicated sepsis was definitely or probably identified, 44% (*n* = 17/39) indicated sepsis might or might not have been identified, 20% (*n* = 8/39) indicated the huddles definitely or probably did not identify sepsis, and 10% (*n* = 4/39) did not respond to this question.

The primary barrier identified by 82% (*n* = 32/39) of providers was the bedside huddle, which was usually triggered after the team already recognized sepsis and initiated treatment. The major facilitator to the alert system and bedside huddle was that, even though the alert often fired after sepsis was identified, the bedside huddle nevertheless resulted in changes in patient care management 44% (*n* = 17/39) of the time.

Overall, 74% (*n* = 29/39) of providers agreed the bedside huddle resulted in a likelihood of performing an intervention. When the huddle yielded a change, most (51%; *n* = 20/39) cited they needed to consult with the fellow and/or attending before making a change to the care of their patient. Only 44% (*n* = 17/39) of providers indicated they probably or definitely wrote a follow-up clinical note prompted by the huddle.

### Targets for future countermeasures

Multiple gaps in interpreting data from the 3-part automated sepsis alert, nurse sepsis screen, and sepsis huddle process were uncovered in this evaluation as targets for potential future countermeasures (Table 1). Some of these identified gaps include the need for (1) medical team education and reeducation on both pSSC and IPSO sepsis guidelines as well as specifics related to the sepsis alert process implemented within the hospital and the PICU, (2) improvements in technology such that the alert system does not cause inefficiencies at the bedside, and (3) refinement of the sepsis alert so that it does not fire after the patient has been identified as having septic shock.

**Table 1.** Identified gaps and targets for potential countermeasures, grouped by themes.

Themes	Identified gaps
Educational deficits	**Process in pediatric intensive care unit** Medical Doctor/Nurse practitioners do not consistently attend bedside huddle Note not written after huddle completion No accountability/feedback on completion of the sepsis huddle protocol, including documentation**Knowledge deficit** Unaware of pediatric Surviving Sepsis Campaign 2020 No Clinical Practice Guideline or sepsis bundle Recognition of deteriorating patients by frontline providers Re-evaluation after sepsis diagnosis Unaware of Sepsis Order Set Not confident in diagnosis or sepsis or septic shock No pocket cards for reference
Technologic deficits	**EMR-Health Information Technology Services related** Patients not being identified appropriately by screen Primary screens trigger after sepsis and/or septic shock identified Already treating sepsis prior to screen trigger No mortality prediction (pSOFA) Functional time zero not part of the protocol, nor tracked Not all primary screens lead to secondary huddle^a^ Alert responses do not correlate with options on screen used by bedside nursing (eg, no option for already treating sepsis or end of life care)^a^**EMR user related** RN activity link/override, opting out of completion of screening Respiratory therapy treatments triggering primary screen Number of primary screen fires too frequently^a^ Repeated pages to charge nurses**Documentation** Severe Sepsis/Septic Shock not documented (ICD-10 codes) Bedside huddles are not identifying severe sepsis or septic shock Unable to determine actions at huddle**Data gathering** No benchmark data Not tracking trigger number at which primary screen fires^a^ Data across multiple spreadsheets^a^
Patient safety considerations	**Delay in care** Length of time medications take to arrive at bedside Delayed interventions (eg, fluid bolus) Awaiting additional consulting service recommendations prior to treatment Frontline providers too busy to complete screens

a denotes remaining gaps. pSOFA = pediatric sequential organ failure assessment. Functional time zero is defined by CHA IPSO as intent to treat sepsis.

## Discussion

Our in-depth review of the implementation of severe sepsis and septic shock screening and identification process in Mott Children’s Hospital PICU demonstrated primary automated sepsis alerts were triggered more than twice as many times as desirable, and often after severe sepsis had already been identified by the bedside clinicians—risking alarm fatigue and adding inefficiencies to time-sensitive patient management.

The purpose of an automated sepsis alert is to be highly “sensitive” to identify nearly all patients possibly with sepsis and miss very few. However, we found routine respiratory therapy treatments prompted ∼15% of sepsis alerts. An excessive number of follow-up alerts were found, some of which were generated by the bedside nurse not properly completing the secondary screen as required by the EMR user interface. Unless the nurse screen form is completed as designed, the primary alert will not silence or suppress, contributing to more alarm fatigue.

Our sepsis team continues to work on mitigating inappropriate sepsis alerts in the critical care environment, highlighting the challenge and need to continually review sepsis alert algorithms and trigger sensitivity.^11^ Potential harms of an automated sepsis screen, importantly, include alert fatigue.^12^ This may lead to the possibility of bedside nurses closing or skiping the primary alert inappropriately. Secondly, despite extensive background work, our data suggest our original PICU threshold may need further refinement both in determining appropriate cutoffs and in selecting data inputs to the trigger. This finding is not unique,^11^ particularly when trying to settle on appropriate alert thresholds in critical care, rather than in an acute care environment.^12^

Survey results from our end users suggested the bedside huddle often resulted in multi-disciplinary communication which enabled appropriate and timely management changes—a clear benefit. Identification of areas to be improved by end users, such as pharmacy delivery time and administration of correct antibiotics, can have a significant impact on compliance with the pSSC guidelines and the timing of the sepsis screen and subsequent interventions. It is essential to incorporate feedback from end-users and continually assess and improve the sepsis screening system to ensure optimal patient outcomes. Since this project began, both our department and our division have applied additional personnel and technology-based resources to address our identified barriers.

Multidisciplinary sepsis education curricula is key to boosting knowledge and attitudes among pediatric providers.^13^^,^^14^ We found only half of medical providers were confident in sepsis diagnosis. As a training institution, we must further optimize educational opportunities to improve sepsis identification and management.

Our data underscored the need for improved and integrated technology to create a more accurate sepsis alert system with more meaningful interactivity with the bedside user. Interactive, EMR-based clinical decision support systems are still relatively new in practice.^13^^,^^14^ If improperly applied, clinical decision support tools can yield patient management delays, thereby impacting patient safety.^11^ It will be important for our EMR and analytics teams to track the sepsis screening tool entirely from start to finish, including tracking the number of primary sepsis alerts and whether the screen leads to a bedside huddle.

Our survey results echoed the sheer number of primary alerts as a potential patient safety concern. For example, the primary alert triggered immediately upon arrival of a critically ill patient from the emergency department or acute care ward. Additionally, primary alerts have fired during a code situation and altering the ability of the bedside nurse to document properly. A third scenario was an inability to silence the alert during end-of-life proceedings. These examples highlight the need to adjust to sepsis alert triggering scenarios effectively and promptly when glaring evidence of inappropriate firing is discovered.

This investigation of barriers and facilitators to a previously implemented process had several limitations. First, as a single-center investigation, our results may be less generalizable than multicenter investigations. While our Epic-based trigger system is unique to Mott Children’s Hospital, the gaps and pitfalls we have described should be taken into consideration for any institution undertaking the development of a new electronic health record-based sepsis screening tool. Second, before January 2020, the Mott IPSO Taskforce data analysis did not include bedside huddle categorizations of sepsis status.^15^ Therefore, IPSO-specific sepsis status categorization is only available for Cohort 2 and is not shown here. Third, as with many other institutions, the COVID-19 pandemic diverted personnel and resources from this effort, impacting our ability to adjust the sepsis alert system between Cohort 1 and Cohort 2. Fourth, the trigger value which results in the primary alert is not documented within the EMR, nor is it easily calculable on manual chart review, thus making it impossible to confirm that the primary sepsis alert was initiated at the appropriate threshold nor allowing us to determine which components were responsible for the alert firing at that moment. Finally, the unequal numbers between the primary alert, secondary screen, and the bedside huddle result from improper completion of the screening process. The repeat alerts make it difficult to track which primary alerts generated the secondary screen. Therefore, compliance with bedside huddles was unable to be confirmed given the abundance of additional primary screens and lack of documentation, especially from the bedside huddles.

Pediatric early sepsis identification pathways are the recommended standard for monitoring patients for sepsis.^3^ Adherence to and proper implementation of the pathway has been a key driver for success.^16^^,^^17^ In the wake of our systematic evaluation of this process, and in addition to making necessary technical improvements, our Department has added a Sepsis Coordinator role. Our group has demonstrated the ability to implement specific pSSC recommendations, highlighting the ability to successfully augment this process.^18^ Our assessment of gaps allows us to generate countermeasure to be employed as part of subsequent iterations of the sepsis alert system in the PICU. Some future directions include: analyzing the primary screen to decrease the volume of alerts with particular attention to the “false” primary alerts resulting from the administration of respiratory therapy-related treatments, refinement of the sensitivity and specificity of the primary trigger alert value, improving the use of the sepsis order set and the completion of the bedside huddle note, and refining the initial and subsequent education to all team members about pSSC campaign guidelines, the IPSO process, the component steps of the sepsis alert tool.

## Conclusion

Our experience and evaluation have identified barriers and facilitators to aid other institutions in initiating or improving their sepsis screening process. Our evaluation of EMR-based sepsis screening demonstrated that refinement of the primary alert tool is needed. However, clinicians found the bedside sepsis huddle to be value-added for impacting patient care. Our data suggest that successful implementation of EMR-based sepsis screening tools requires countermeasures that focus on 3 key drivers for change: education, technology, and patient safety considerations. As many institutions across the world are concurrently developing and refining sepsis screening tools, identification of pearls and pitfalls in sepsis screening methodology can advance rapid, widespread implementation of sepsis screening tools and, thus, early recognition of sepsis.

## Data Availability

The data underlying this article will be shared on reasonable request to the corresponding author.

## References

[1] Weiss SL , Fitzgerald JC, Pappachan J, et al; Sepsis Prevalence, Outcomes, and Therapies (SPROUT) Study Investigators and Pediatric Acute Lung Injury and Sepsis Investigators (PALISI) Network. Global epidemiology of pediatric severe sepsis: the sepsis prevalence, outcomes, and therapies study. Am J Respir Crit Care Med. 2015;191(10):1147-1157. 10.1164/rccm.201412-2323OC 25734408 PMC4451622

[2] What is sepsis? 2022. Sepsis alliance. Retrieved from the World Wide web on August 2022 at: http://www.sepsis.org/sepsis-basics/what-is-sepsis

[3] Weiss SL , Peters MJ, Alhazzani W, et al Surviving sepsis campaign international guidelines or the management of septic shock and sepsis-associated organ dysfunction in children. Intensive Care Med. 2020;46(S1):10-67. 10.1007/s00134-019-05878-6 32030529 PMC7095013

[4] Prusakowski MK , Chen AP. Pediatric sepsis. In: Emergency Medicine Clinics of North America. 2017. 10.1016/j.emc.2016.08.008 27908329

[5] Children’s Hospital Association. 2020. Improving pediatric sepsis outcomes (IPSO) is successfully challenging sepsis. Retrieved from the World Wide Web on March 2020 at: http://www.childrenshospitalassociation.org

[6] Eisenberg MA , Balamuth F. Pediatric sepsis screening in US hospitals. Pediatr Res. 2022;91(2):351-358. 10.1038/s41390-021-01708-y 34417563 PMC8378117

[7] Goldstein B , Giroir B, Randolph A; International Consensus Conference on Pediatric Sepsis. International pediatric sepsis consensus conference: definitions for sepsis and organ dysfunction in pediatrics. Pediatr Crit Care Med. 2005;6(1):2-8. 15636651 10.1097/01.PCC.0000149131.72248.E6

[8] Pediatric advanced life support vital signs. 2020. Retrieved from the World Wide Web on March 2020 at: http://cpr.herat.org

[9] Scott HF , Brilli RJ, Paul R, et al Evaluating pediatric sepsis definitions designed for electronic health record extraction and multicenter quality improvement. *Crit Care Med*. 2020;48(10):e916-e926. 10.1097/CCM.0000000000004505 PMC7677030 32931197

[10] Qualtrics. 2020. Provo, Utah, USA. http://www.qualtrics.com

[11] Bradshaw C , Goodman I, Rosenberg R, Bandera C, Fierman A, Rudy B. Implementation of an inpatient pediatric sepsis identification pathway. Pediatrics. 2016;137(3):e20144082. 10.1542/peds.2014-4082 26908676

[12] Eisenberg MA , Balamuth F. Pediatric sepsis screening in US hospitals. Pediatr Res. 2021;91(2):351-358. Advanced online publication. 10.1038/s41390-021-01708-y 34417563 PMC8378117

[13] Shivers L , Feldman SS, Hayes LW. Development of a computerized paediatric intensive care unit septic shock pathway: improving user experience. Health Syst (Basingstoke). 2019;8(3):155-161. 10.1080/20476965.2019.1620638 31839928 PMC6896506

[14] Breuer RK , Hassinger AB. Impact of a multidisciplinary sepsis initiative on knowledge and behavior in a pediatric center. Pediatr Qual Saf. 2020;5(2):e267. 10.1097/pq9.0000000000000267 32426633 PMC7190264

[15] Sutton RT , Pincock D, Baumgart DC, Sadowski DC, Fedorak RN, Kroeker KI. An overview of clinical decision support systems: benefits, risks, and strategies for success. NPJ Digit Med. 2020;3(1):17. 10.1038/s41746-020-0221-y 32047862 PMC7005290

[16] Depinet H , Macias CG, Balamuth F, et al; American Academy of Pediatrics Pediatric Septic Shock Collaborative (PSSC) Investigators. Pediatric septic shock collaborative improves emergency department sepsis care in children. Pediatrics. 2022;149(3):e2020007369. 35229124 10.1542/peds.2020-007369

[17] Lane RD , Funai T, Reeder R, Larsen GY. High-reliability pediatric septic shock quality improvement initiative and decreasing mortality. Pediatrics. 2016;138(4):e20154153. 10.1542/peds.2015-4153 27604184

[18] Mazloom A , Sears SM, Carlton EF, Bates KE, Flori HR. Implementing pediatric surviving sepsis campaign guidelines: improving compliance with lactate measurement in the PICU. Crit Care Explor. 2023;5(4):e0906. 37101534 10.1097/CCE.0000000000000906 PMC10125524

